# Supplementary figures and images for: FUN-PROSE: A deep learning approach to predict condition-specific gene expression in fungi
Source: PLoS Comput Biol. 2023 Nov 16;19(11):e1011563. doi: 10.1371/journal.pcbi.1011563 (PMC10653424; doi:10.1371/journal.pcbi.1011563)

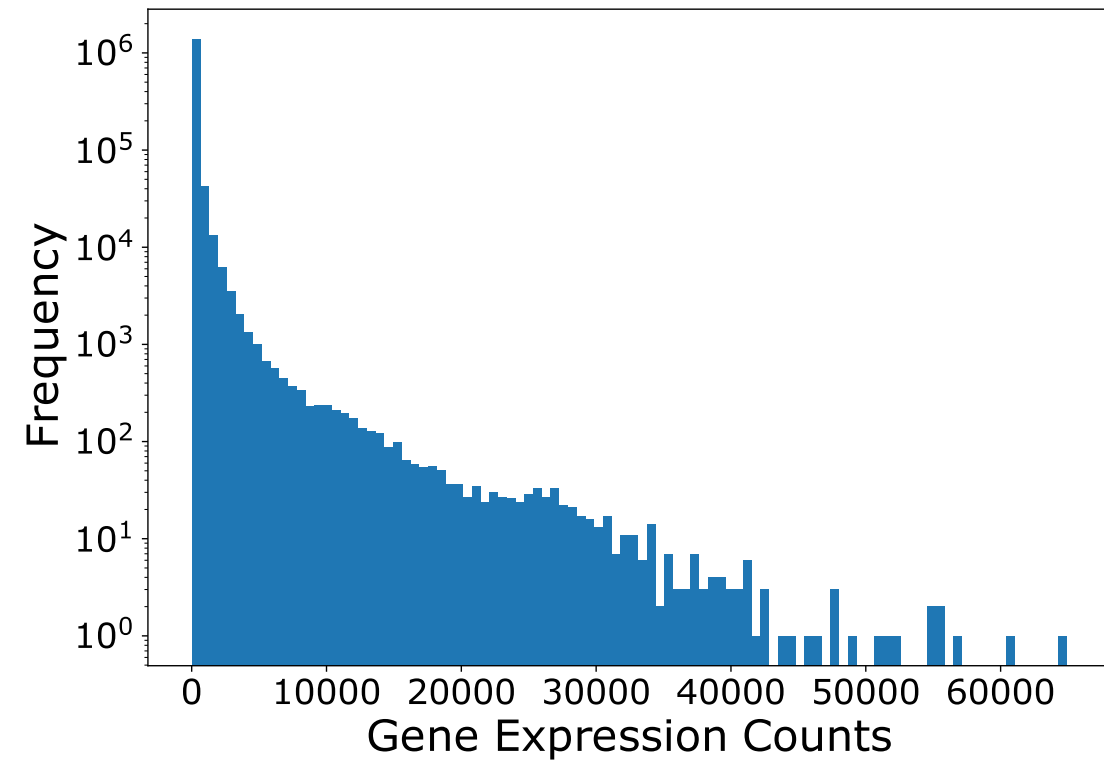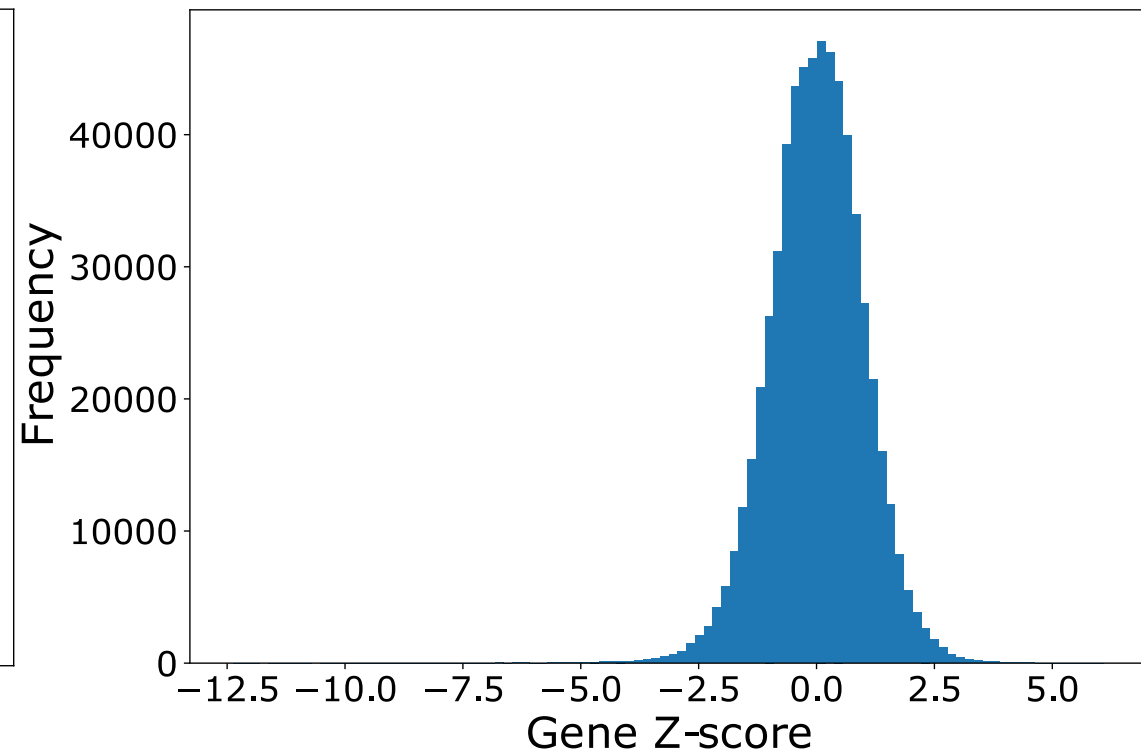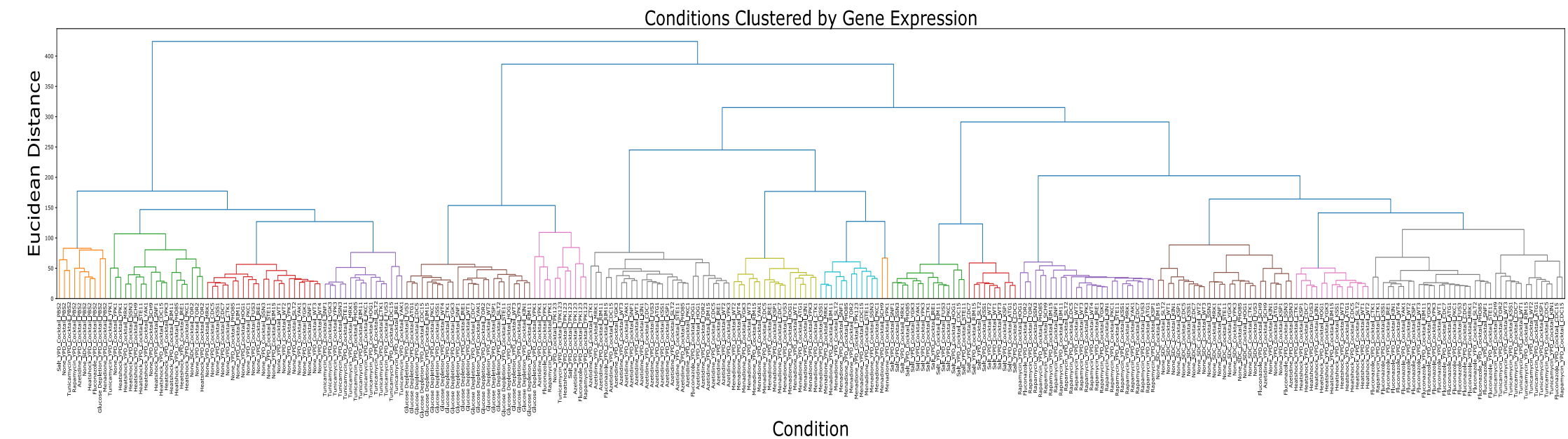

Supplement: S1 Fig — (Top-left) Histogram of gene expression with CV filter. (Top-right) Histogram of Z-scored expressions. (Bottom) The different conditions in the dataset clustered by gene expression using agglomerative clustering. (PDF) [file pcbi.1011563.s001.pdf]

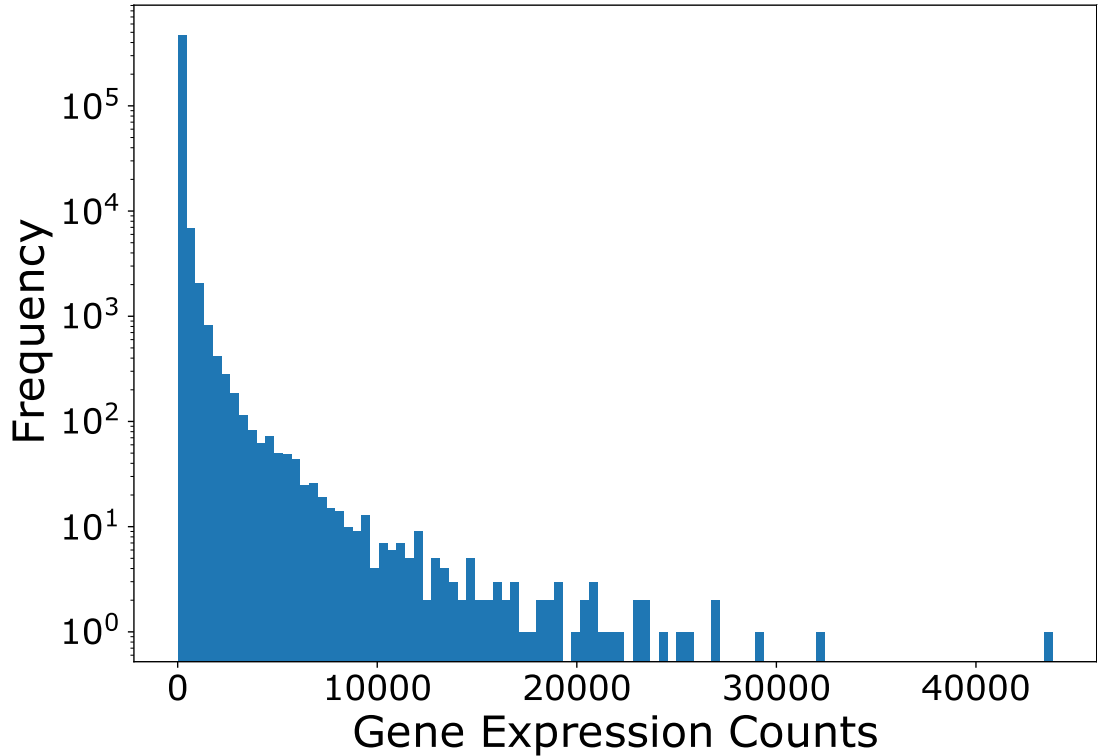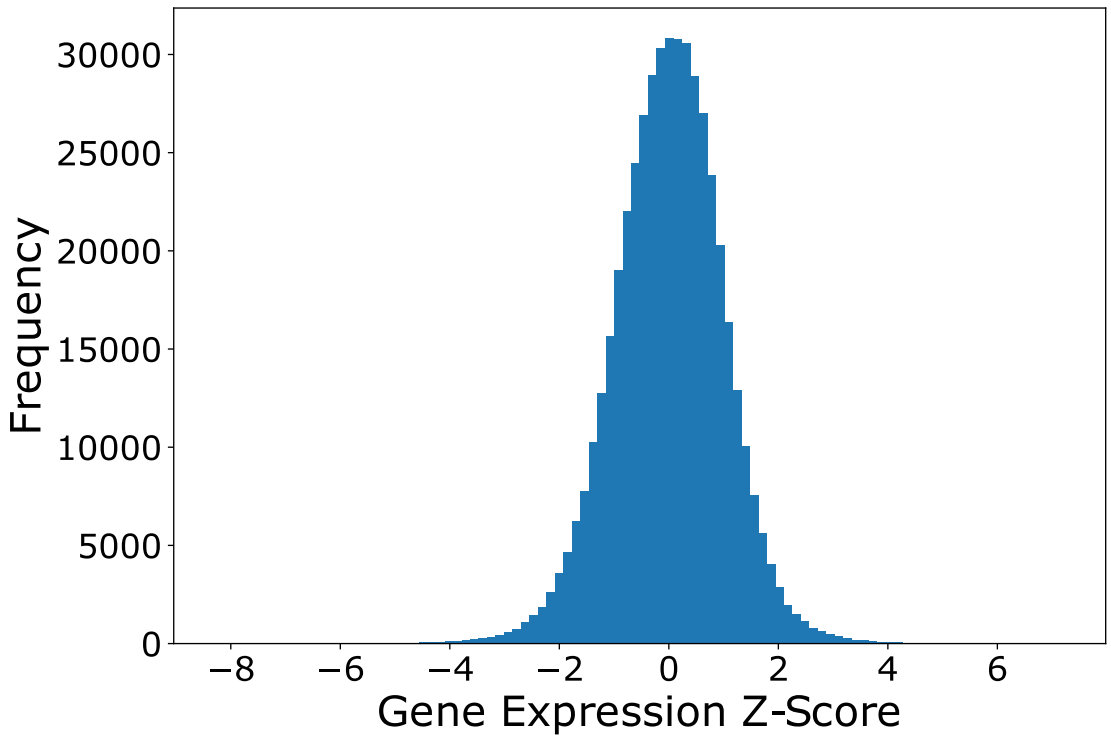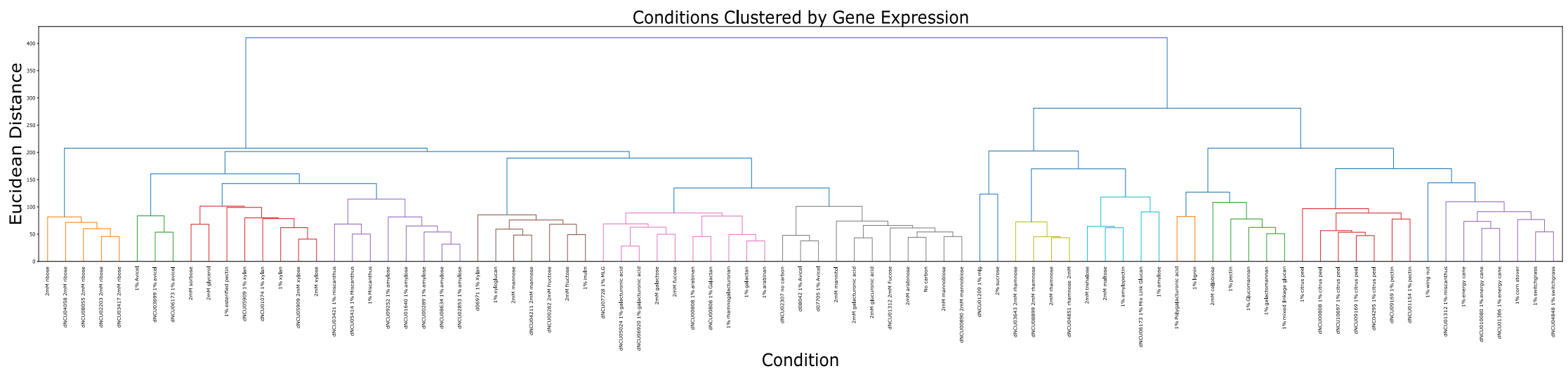

Supplement: S2 Fig — (Top-left) Histogram of gene expression with CV filter. (Top-right) Histogram of Z-scored expressions. (Bottom) The different conditions in the dataset clustered by gene expression using agglomerative clustering. (PDF) [file pcbi.1011563.s002.pdf]

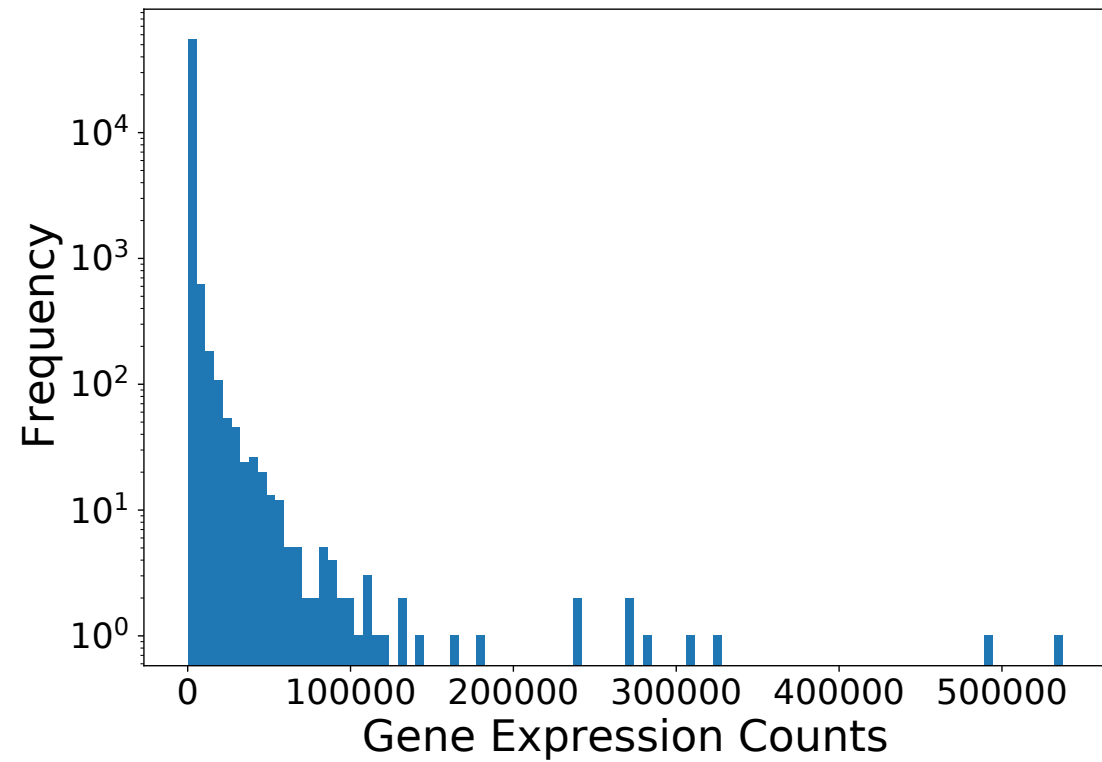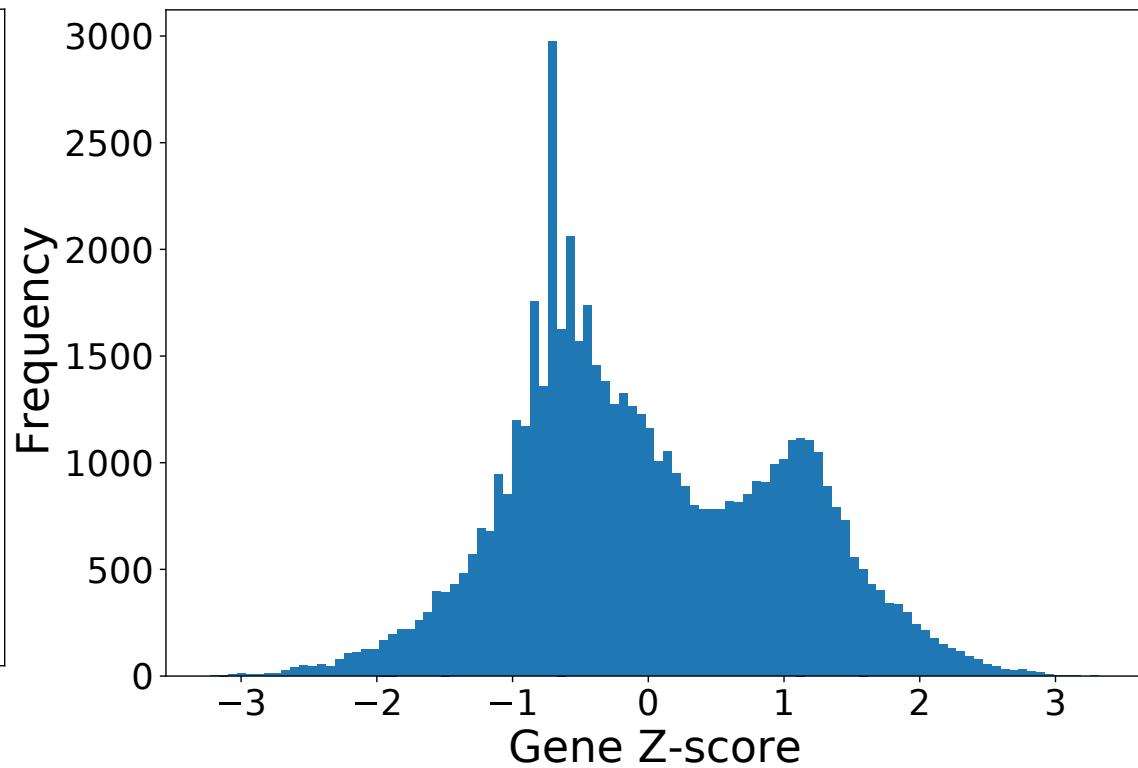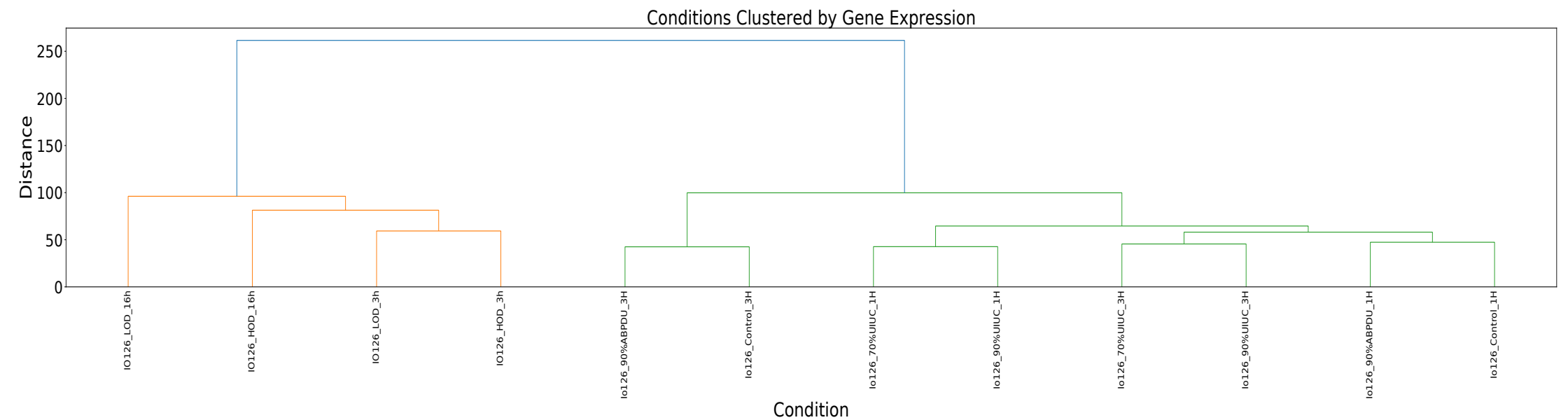

Supplement: S3 Fig — (Top-left) Histogram of gene expression with CV filter. (Top-right) Histogram of Z-scored expressions. (Bottom) The different conditions in the dataset clustered by gene expression using agglomerative clustering. (PDF) [file pcbi.1011563.s003.pdf]

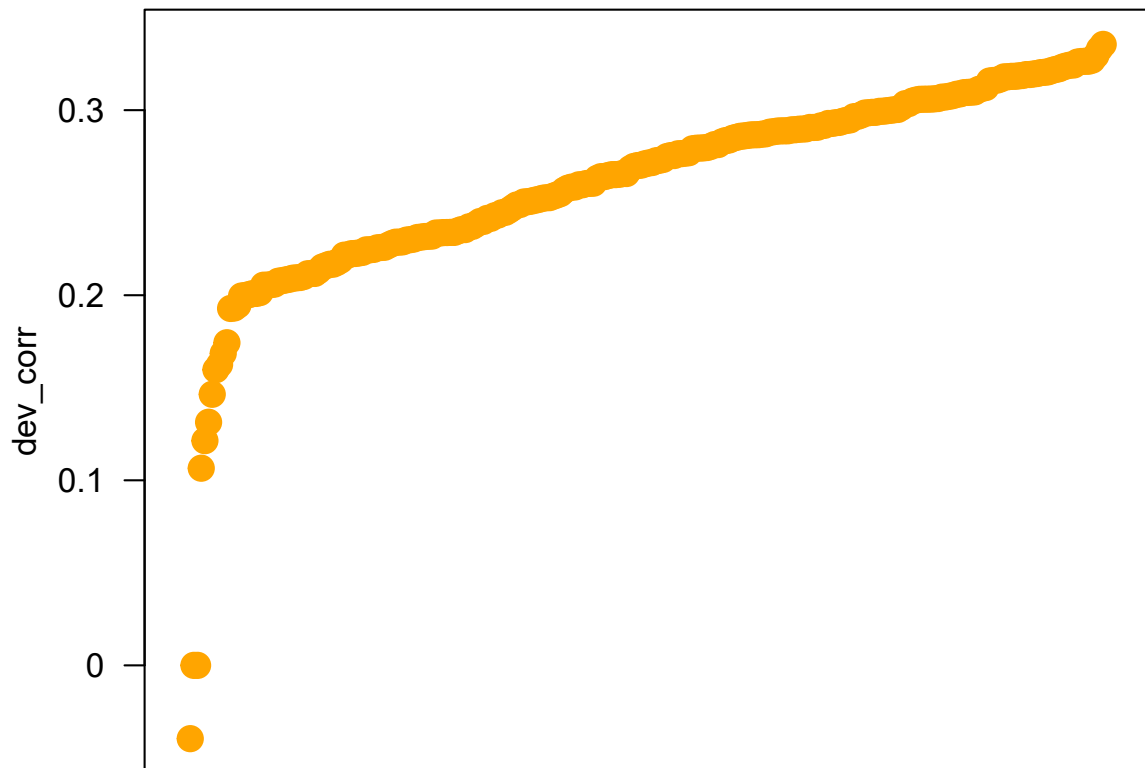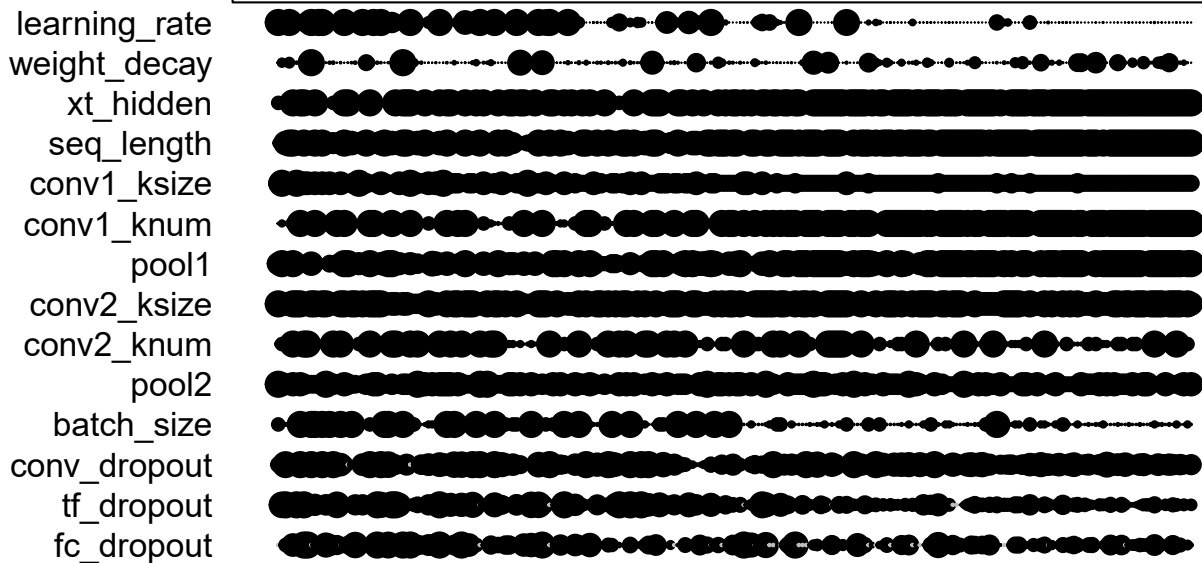

Supplement: S4 Fig — The y-axis of the plot shows the correlation between predicted and measured expression for the validation set. The trials on the plot are sorted in ascending correlations. On the x-axis, we show the various hyperparameters that we are optimizing. The size of the black markers represent the value of the hyperparameter for that trial. For example, this plot can be interpreted to show that a smaller learning rate leads to better performance. (PDF) [file pcbi.1011563.s004.pdf]

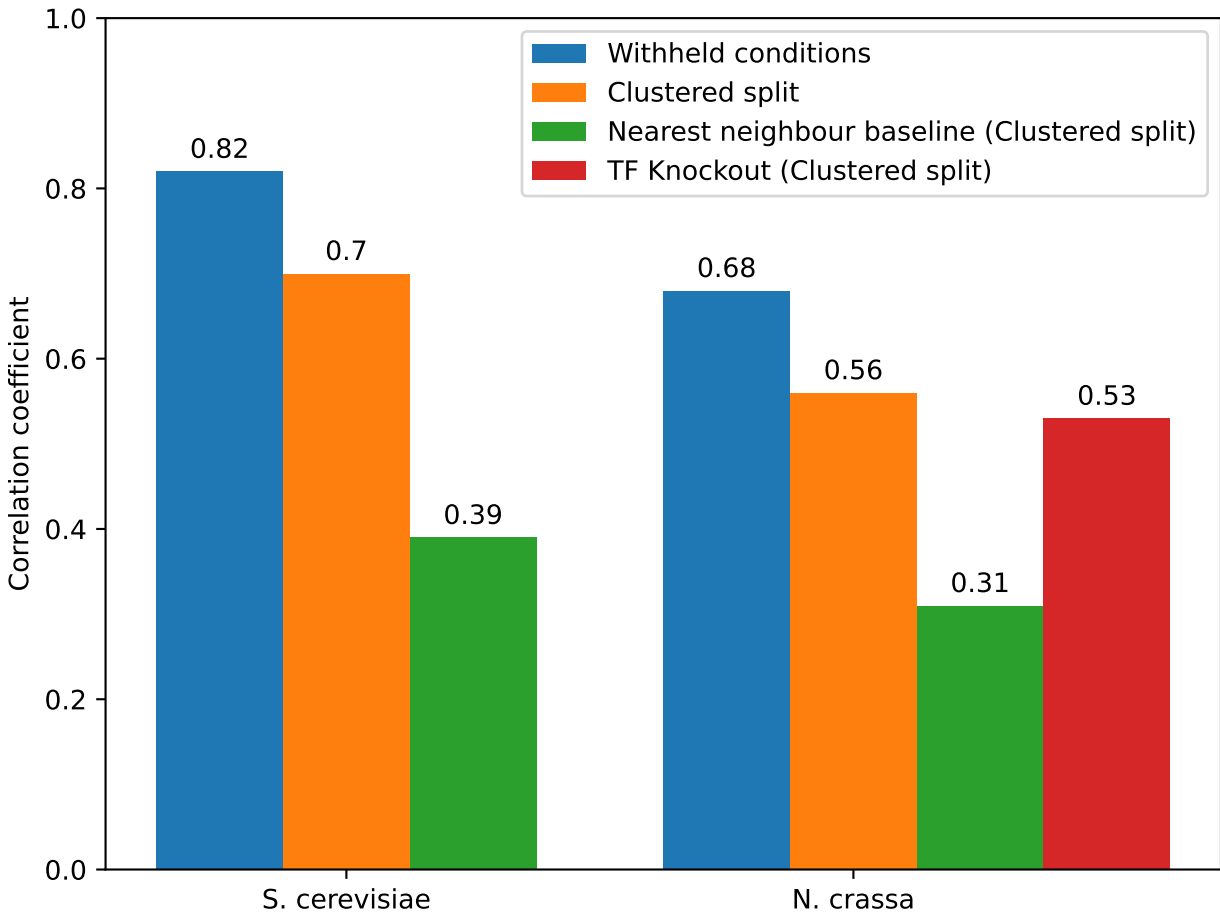

Supplement: S5 Fig — The performance of FUN-PROSE on the withheld conditions split (in blue) is compared to a more rigorous split (clustered split; in orange). To generate the clustered split, the conditions are clustered using hierachical clustering, as shown on S1 and S2 Figs. Then, the train and test splits are generated such that no condition in found in the train split is found in the test split, and vice-versa. The performance of FUN-PROSE is then compared to a simpler Nearest Neighbor Regression model (in green). Finally, FUN-PROSE is used to predict the effects of TF knockout (shown in red). (PDF) [file pcbi.1011563.s005.pdf]

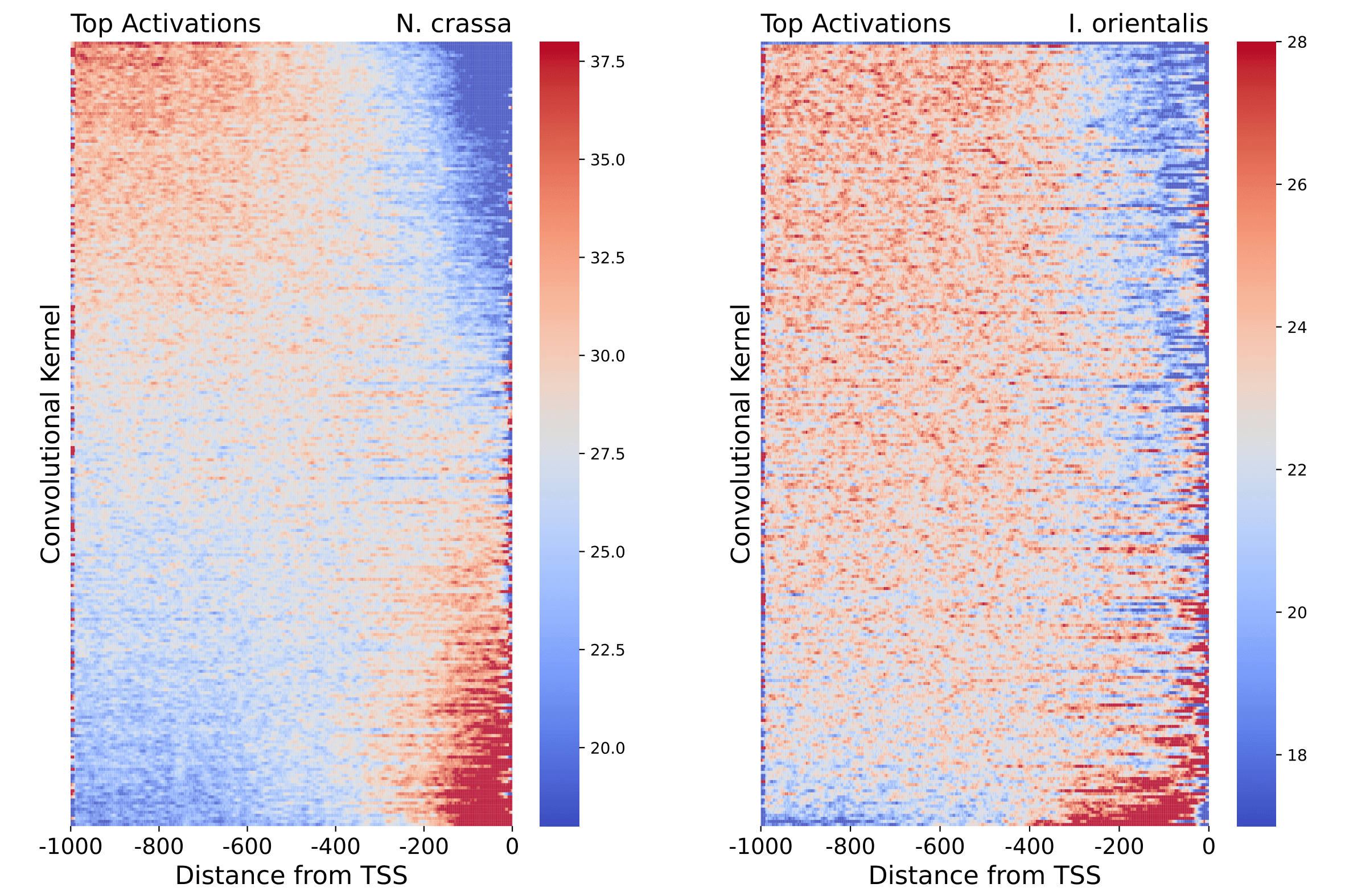

Supplement: S6 Fig — The rows of this heatmap are sorted by the average activation level within 300bp from the transcription start site. Note that most motifs exhibit non-random positional preferences indicative of biological function. (PNG) [file pcbi.1011563.s006.png]

**a**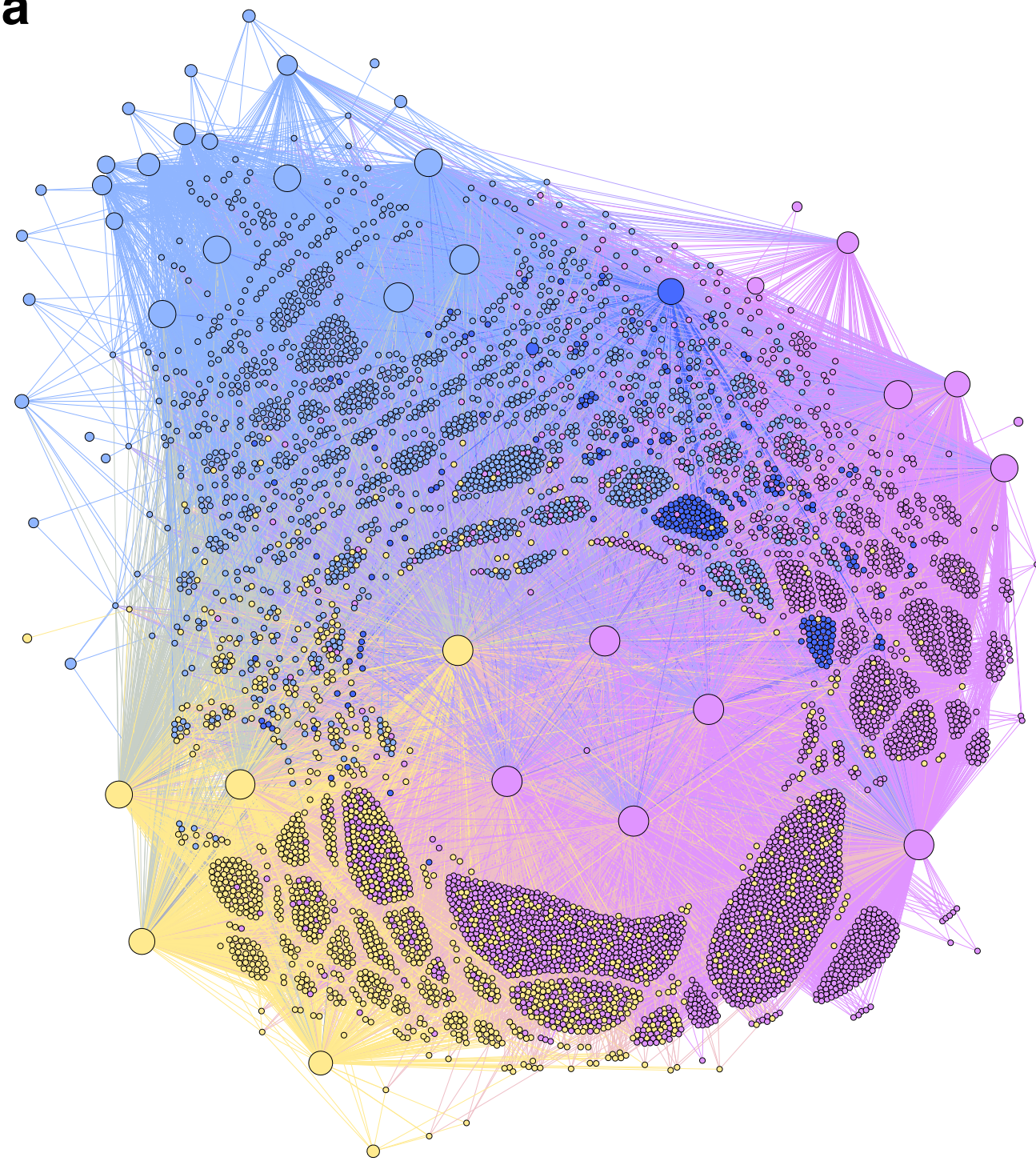**b**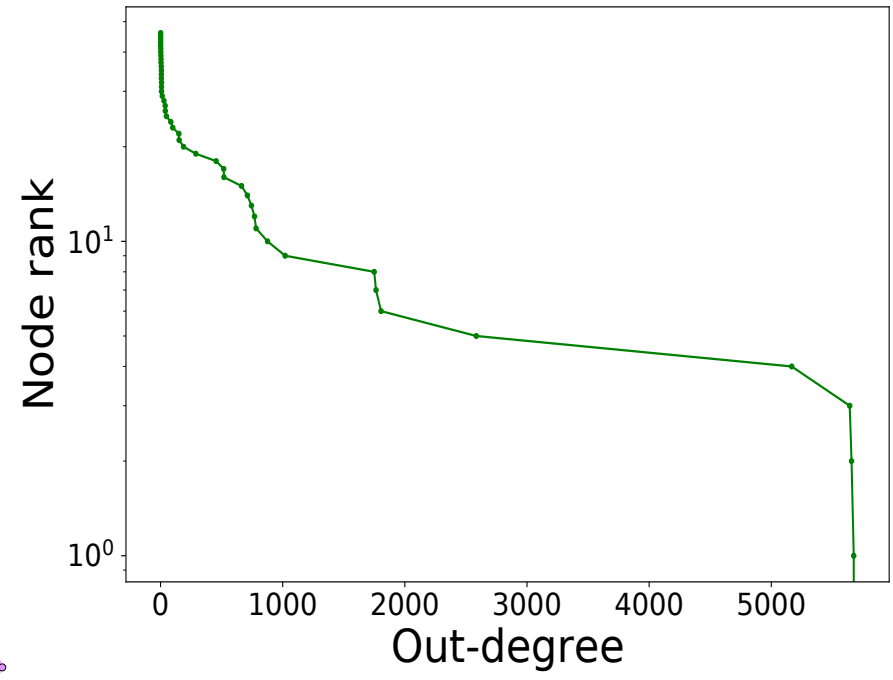**c**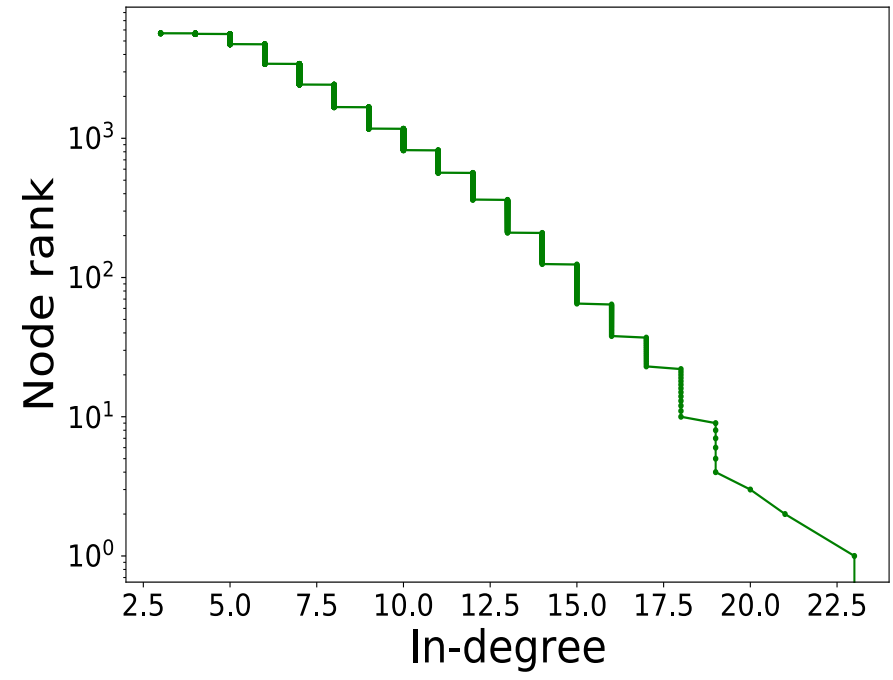

Supplement: S7 Fig — (a) The network of TF-target gene interactions obtained by applying a 2.5 standard deviation threshold to the TF-target gene Integrated Gradients scores for the N. crassa dataset. Nodes are colored by clusters obtained by modularity analysis. Node sizes are proportional to their out-degree. (b) Cumulative histogram (number of nodes with degree > = x) of out-degrees of TFs and (c) in-degrees of target genes. (PDF) [file pcbi.1011563.s007.pdf]

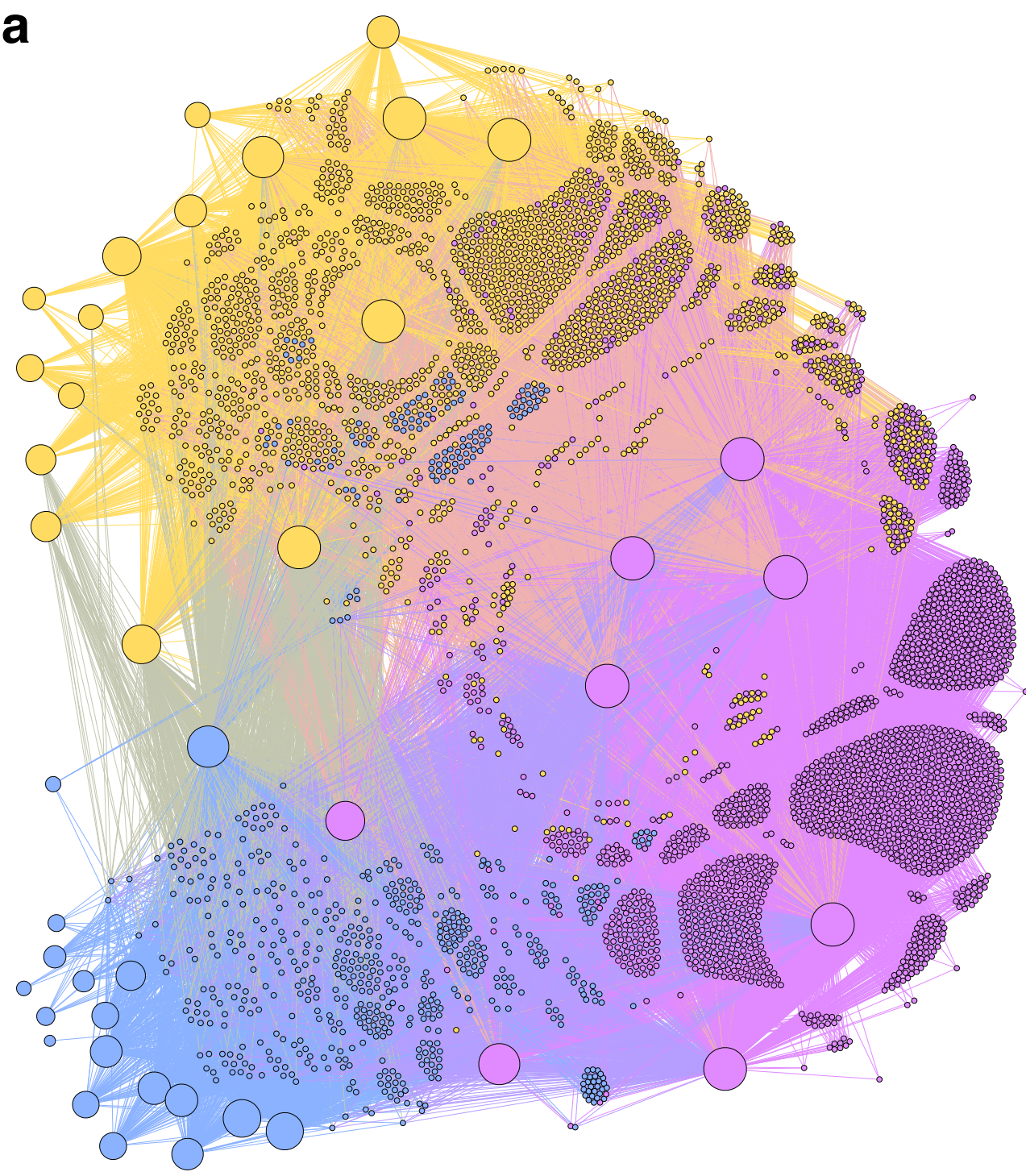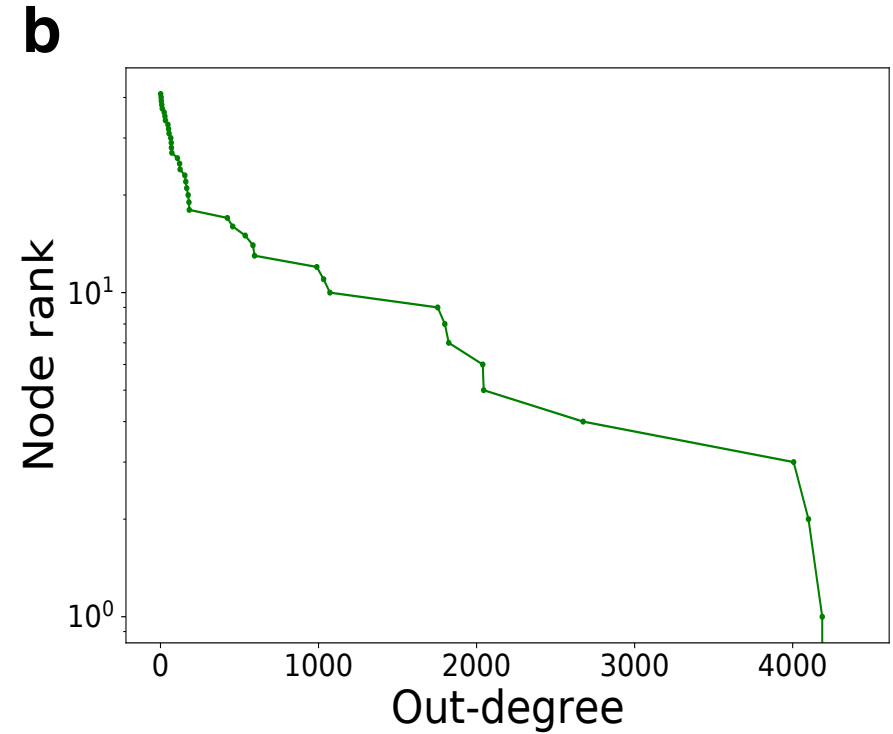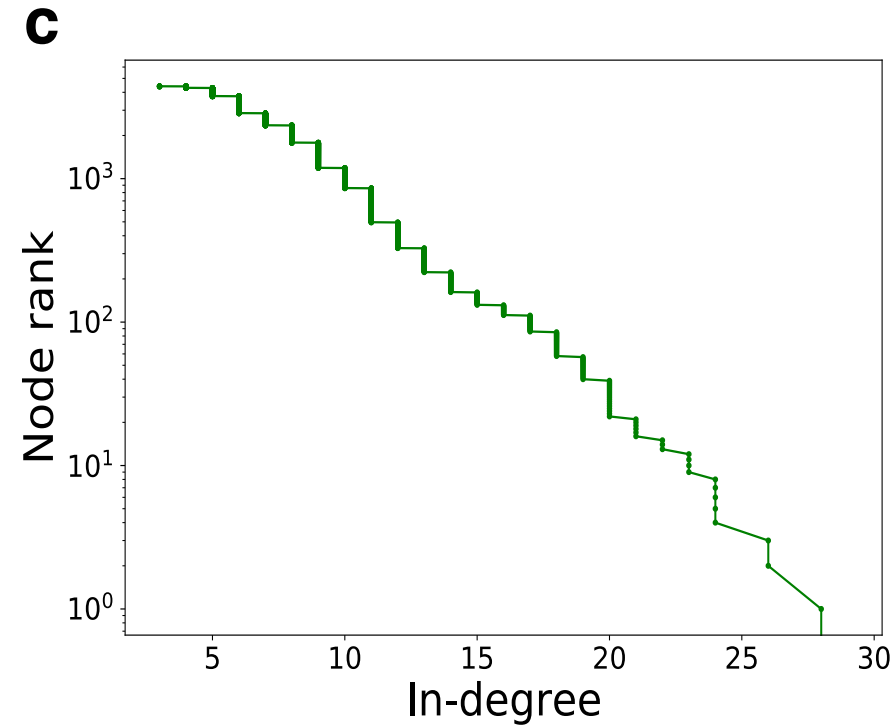

Supplement: S8 Fig — (a) The network of TF-target gene interactions obtained by applying a 2 standard deviation threshold to the TF-target gene Integrated Gradients scores for the I. orientalis dataset. Nodes are colored by clusters obtained by modularity analysis. Node sizes are proportional to their out-degree. (b) Cumulative histogram (number of nodes with degree > = x) of out-degrees of TFs and (c) in-degrees of target genes. (PDF) [file pcbi.1011563.s008.pdf]

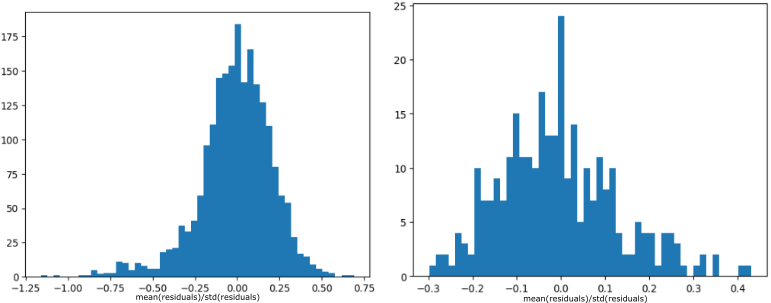

Supplement: S10 Fig — The distribution of the mean residuals divided by standard deviation of residuals for each gene (left) and for each condition (right) in the S. cerevisiae test data. (PNG) [file pcbi.1011563.s010.png]

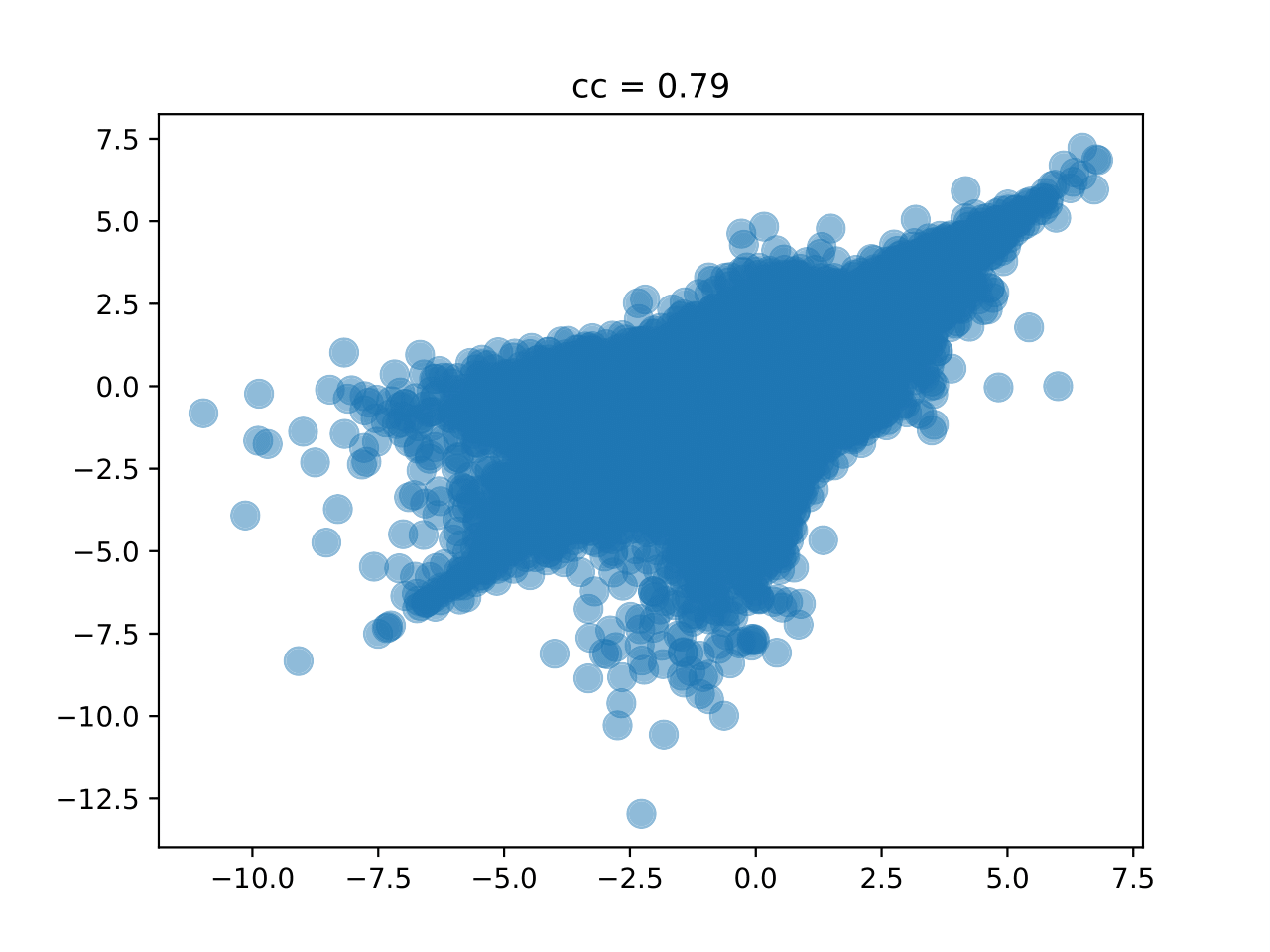

Supplement: S11 Fig — This is done using a scatter plot of replicates 1 and 2 of the data which have a Pearson correlation coefficient of 0.79. (PNG) [file pcbi.1011563.s011.png]

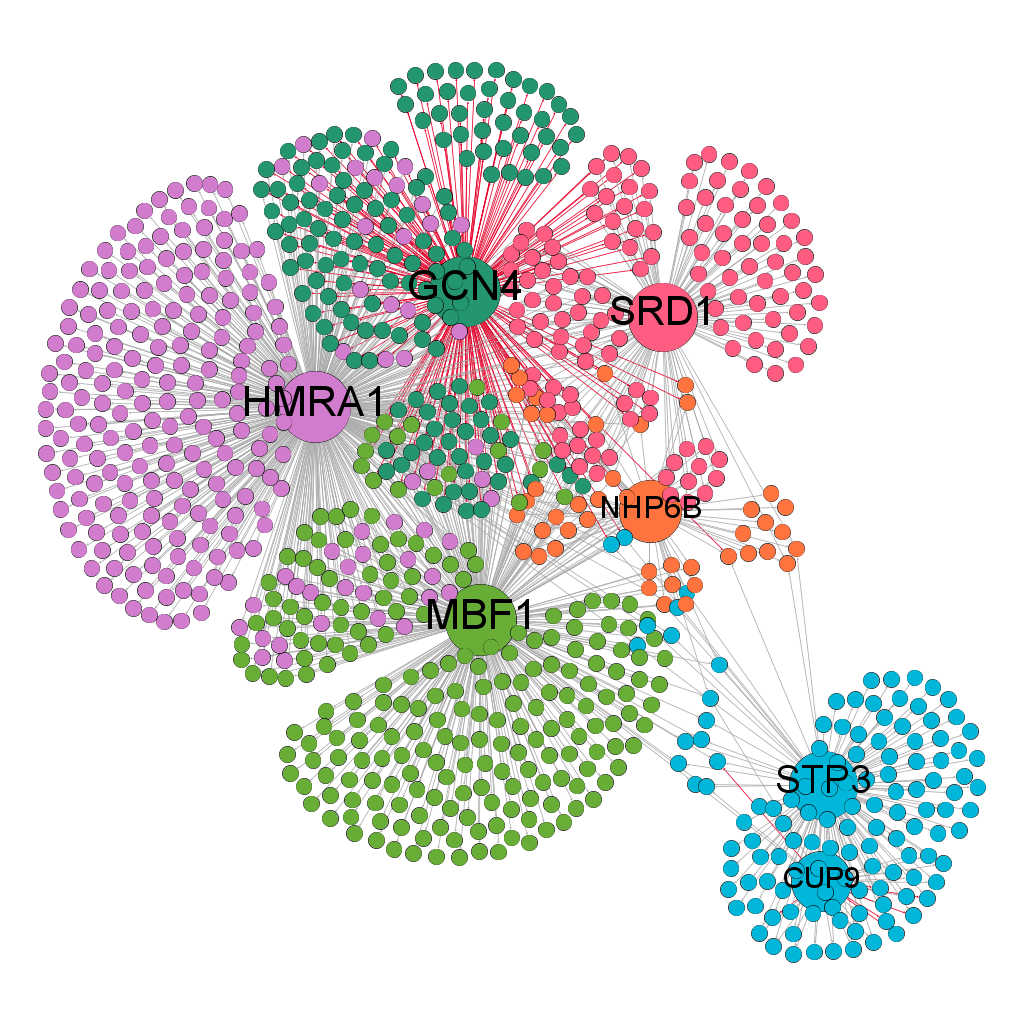

Supplement: S13 Fig — The network from Fig 4A was filtered so that only edges So that each TF-gene link was only kept if the gene was activated by a convolutional filter associated with the TF as listed in S12 Fig. (PNG) [file pcbi.1011563.s013.png]

## Extracted kernel

## Known PSWM in *S.cerevisiae*

## Activation profile

## Known binding in *S.cerevisiae*

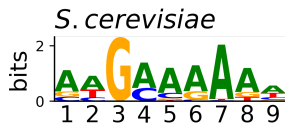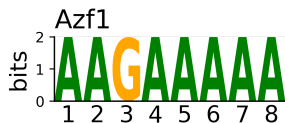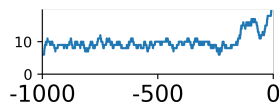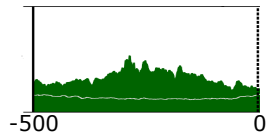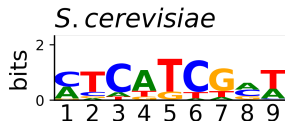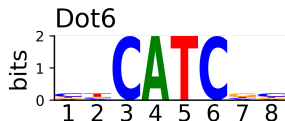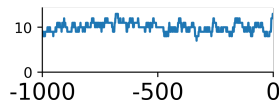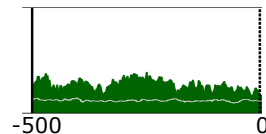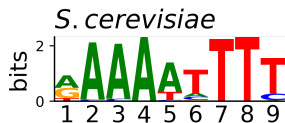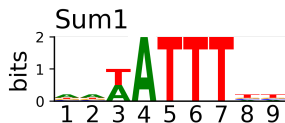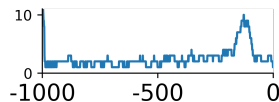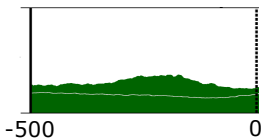

Supplement: S14 Fig — The activation profiles of motifs from Fig 4 are compared to binding locations presented by the Yeast Epigenome Project. (PDF) [file pcbi.1011563.s014.pdf]
