# Supplementary material for: FUN-PROSE: A deep learning approach to predict condition-specific gene expression in fungi
Source: PLoS Comput Biol. 2023 Nov 16;19(11):e1011563. doi: 10.1371/journal.pcbi.1011563 (PMC10653424; doi:10.1371/journal.pcbi.1011563)
Supplement: S9 Fig — (PDF) [file pcbi.1011563.s009.pdf]

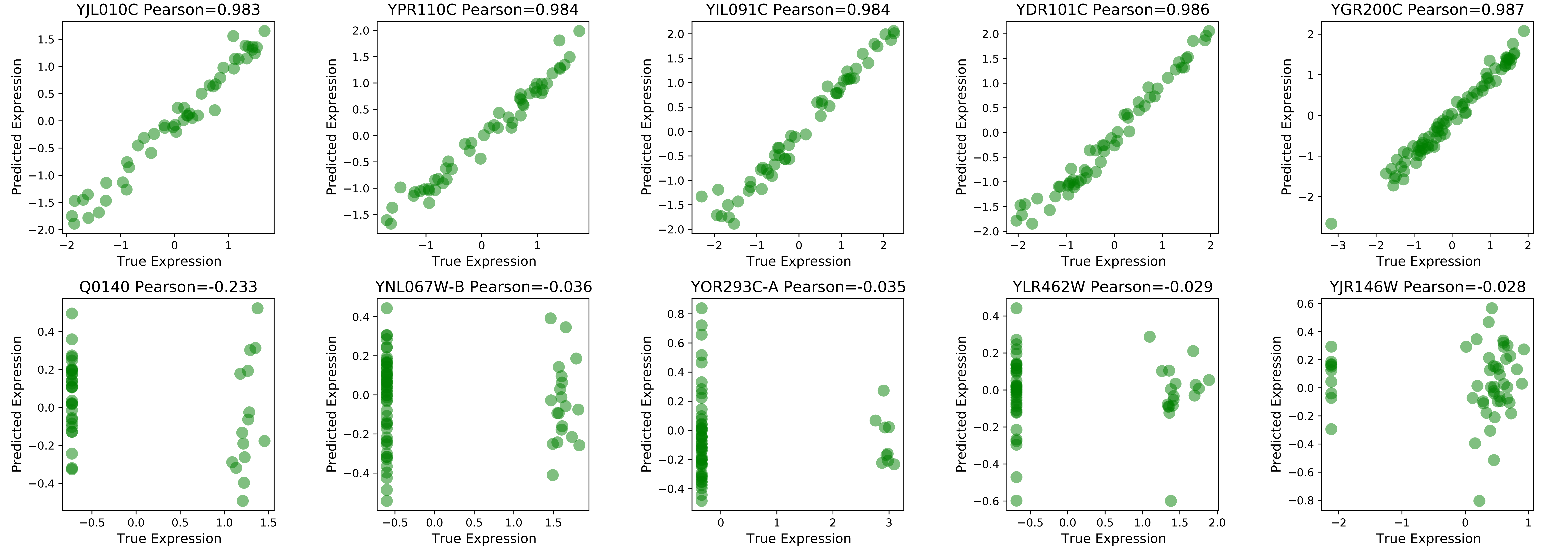

YJL010C Pearson=0.983
YPR110C Pearson=0.984
YIL091C Pearson=0.984
YDR101C Pearson=0.986
YGR200C Pearson=0.987
Q0140 Pearson=-0.233
YNL067W-B Pearson=-0.036
YOR293C-A Pearson=-0.035
YLR462W Pearson=-0.029
YJR146W Pearson=-0.028
Predicted Expression
True Expression
