# Supplementary material for: FUN-PROSE: A deep learning approach to predict condition-specific gene expression in fungi
Source: PLoS Comput Biol. 2023 Nov 16;19(11):e1011563. doi: 10.1371/journal.pcbi.1011563 (PMC10653424; doi:10.1371/journal.pcbi.1011563)
Supplement: S12 Fig — The matching was made by first taking the network shown in Fig 4A. Then, for each combination of TF (only TFs with ≥ 20 targets were considered) and filter, we ran a hypergeometric test to evaluate if the filter activation was over-represented in the TF’s targets. Filter activation was binarized using a threshold of 1 standard deviation from the mean. (PDF) [file pcbi.1011563.s012.pdf]

TF	Filter ID	FDR	Extracted Motif
STP3	155	0.00736	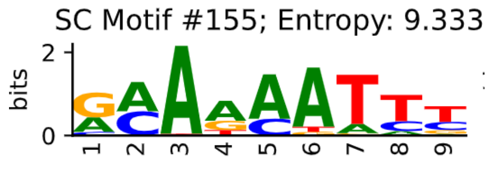
HMRA1	244	0.01251	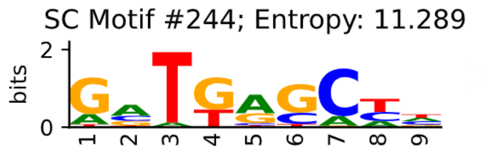
HMRA1	14	0.068802	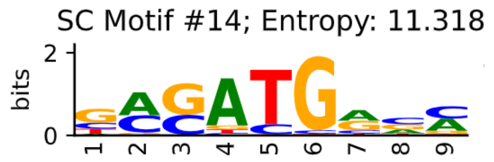
STP3	165	0.053292	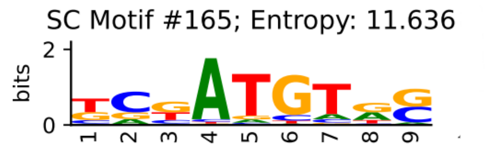
STP3	127	0.045363	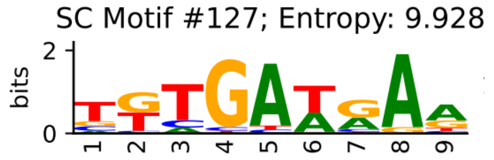
HMRA1	222	0.041268	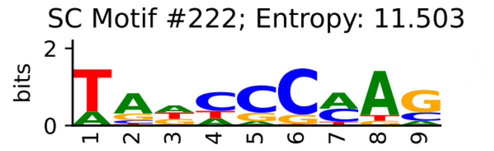
CUP9	165	0.0944	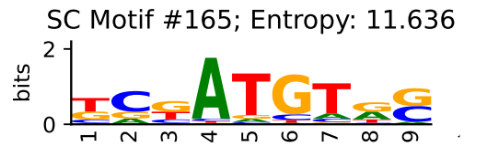
MBF1	167	0.093966	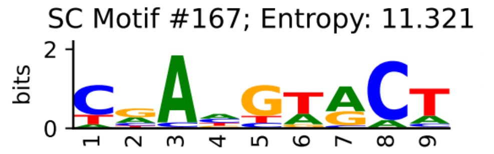
GCN4	14	0.087736	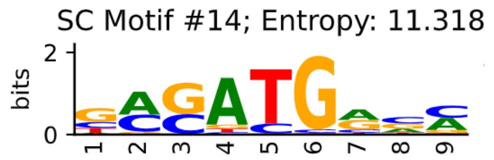
CUP9	155	0.127672	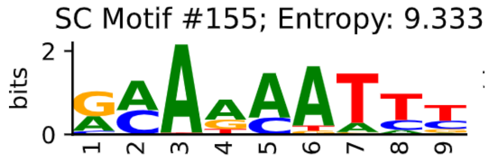
NHP6B	8	0.160799	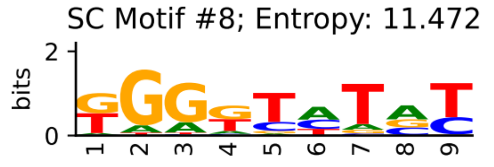
STP3	49	0.186531	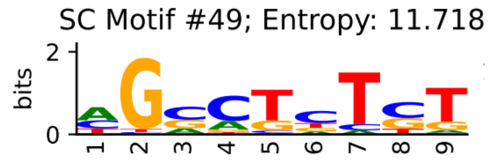
SRD1	94	0.173224	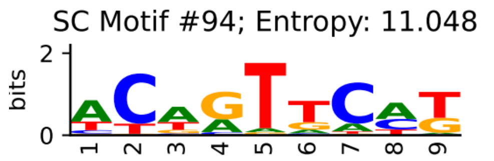
STP3	116	0.18249	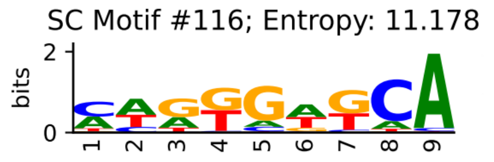
